# Degree of methylation burden is determined by the exposure period to carcinogenic factors

**DOI:** 10.1111/cas.13136

**Published:** 2017-04-03

**Authors:** Hideyuki Takeshima, Tohru Niwa, Takeshi Toyoda, Mika Wakabayashi, Satoshi Yamashita, Toshikazu Ushijima

**Affiliations:** ^1^ Division of EpigenomicsNational Cancer Center Research InstituteTokyoJapan; ^2^ Division of PathologyNational Institute of Health SciencesTokyoJapan

**Keywords:** Aberrant DNA methylation, carcinogenic factor, chronic inflammation, eradication, methylation burden

## Abstract

Aberrant DNA methylation accumulated in normal tissues, namely methylation burden, is associated with risk of carcinogenesis. The levels of methylation burden are known to be influenced by multiple factors, such as genetic factors and strengths of carcinogenic factors. However, the impact of the degree of exposure to a carcinogenic factor is still unclear. Here, using a Mongolian gerbil model of *Helicobacter pylori* (*H*. *pylori*)‐induced gastritis, we aimed to clarify the impact of the degree of exposure on methylation burden in normal gastric tissues. DNA methylation levels of four CpG islands, HE6, SA9, SB5, and SD2, increased by *H*. *pylori* infection, depending upon the infection period. After eradication of *H*. *pylori*, DNA methylation levels decreased, but tended to be higher in gastric mucosae with a longer infection period. DNA molecules with dense methylation, but not those with sparse methylation, increased depending upon the infection period. DNA methylation levels of one of the four CpG islands, SA9, tended to be higher in gastric mucosae of gerbils infected with *H*. *pylori*, even 50 weeks after eradication than in those of non‐infected gerbils. These results showed for the first time that the levels of methylation burden in normal tissues are influenced by the degree of exposure to a carcinogenic factor.

Epigenetic alterations, such as aberrant DNA methylation, are present in normal tissues, and can be causally involved in the development of cancers.^1^, ^2^ To be specific, aberrant DNA methylation at promoter CpG islands of tumor‐suppressor genes and microRNA genes can silence their transcription, similarly to allelic loss.^3^, ^4^ Accumulated DNA methylation in normal tissues, namely methylation burden, is associated with risk of carcinogenesis.^5^ The usefulness of methylation burden in cancer risk diagnosis was recently demonstrated by a large clinical study.^6^ Such aberrant DNA methylation is induced by exposure to carcinogenic factors, such as infectious agents,^7^ tobacco smoking,^8^ and hormones.^9^ Especially, chronic inflammation has been shown to be a strong inducer of aberrant DNA methylation.^10^, ^11^

In gastric tissues, the level of methylation burden is known to be influenced by multiple factors. One is a genetic factor (host factor), namely a single nucleotide polymorphism (SNP) of *IL1B*, which is a known risk factor of gastric carcinogenesis.^12^ DNA methylation levels in gastric mucosae of people with the *IL1B*‐511T/T allele are higher for *CYP1B1* and *GRIN2B* than those of the *IL1B*‐511 C carrier.^13^ Another is the strength of the carcinogenic factor, for example, a pathogenic protein of *Helicobacter pylori* (*H*. *pylori*), Cag‐A. DNA methylation levels in gastric cancers of patients infected with Cag‐A positive *H*. *pylori* are higher than those with Cag‐A negative *H*. *pylori*,^14^, ^15^ suggesting the possible influence of Cag‐A on methylation burden. However, the impact of the degree of exposure (exposure period) to a carcinogenic factor on the methylation burden has not been addressed.

To analyze the impact of the degree of the exposure to a carcinogenic factor, controlled exposure to them and a homogenous genetic background are necessary. From this viewpoint, the Mongolian gerbil model of *H*. *pylori*‐induced gastritis, in which aberrant DNA methylation can be efficiently induced^10^, ^11^ is useful. In this study, using this model, we aimed to clarify the impact of the degree of exposure to a carcinogenic factor on methylation burden.

## Materials and Methods

### Animal experiment

Mongolian gerbils (MGS/Sea; Kyudo, Saga, Japan) were inoculated with *H. pylori* (ATCC43504; ATCC, Manassas, VA, USA) by i.g at 5 weeks of age. Ten, 20, or 50 weeks after infection, *H. pylori* was eradicated by treating gerbils with 3 mg/kg amoxicillin, 10 mg/kg lansoprazole, and 30 mg/kg clarithromycin, as described previously.^11^ At specific periods after eradication, the gerbils were sacrificed, and the stomach was resected. From the resected stomach, gastric epithelial cells were isolated by the gland isolation technique,^16^ and entire gastric tissues containing both gastric mucosae and submucosal layers were also collected. For histological analysis, entire gastric tissue fixed by formalin was embedded into paraffin, and then the formalin‐fixed paraffin‐embedded (FFPE) samples were sliced. The sliced FFPE samples were deparaffinized, and were stained with hematoxylin and eosin. The degree of infiltration of inflammatory cells, namely neutrophils and mononuclear cells, was scored for three of the six (G11), eight (G1, G2, G3, G6, G7, and G10), 12 (G8 and G12), and 15 (G4) gerbils on a four‐point scale (0 to 3), as described previously.^17^ All the animal experiments were approved by the committee for Ethics in Animal Experimentation at the National Cancer Center.

### Cell lines

Two gerbil gastric cancer cell lines, MGC1 and MGC2,^18^ were kindly provided by Dr M. Tatematsu, and were maintained in RPMI1640 medium containing 10% (v/v) FBS.

### Quantitative methylation‐specific PCR

Genomic DNA was extracted from isolated gastric epithelial cells. One microgram of genomic DNA was digested with BamHI, treated with sodium bisulfite, and purified by Zymo‐Spin column I (Zymo Research, Irvine, CA, USA). Purified DNA was dissolved in 40 μL of 1× TE, and 1 μL of the bisulfite‐treated DNA was used for quantitative methylation‐specific PCR (qMSP) using primers listed in Table S1, as described previously.^19^

DNA methylation levels of four CpG islands, HE6 (exon 2 of *Ntrk2* gene), SA9 (exon 1 of *Nol4* gene), SB5 (location not identified), and SD2 (promoter of *Nptx2* gene), which were identified as those aberrantly methylated in gerbil gastric mucosae by *H*. *pylori* infection,^11^ were measured using the percentage of the methylation reference (PMR), which was calculated as [(number of methylated DNA molecules at a target CpG island in a sample)/(number of B2 repeat in the sample)]/[(number of methylated DNA molecules at the target CpG island in a fully methylated DNA sample)/(number of B2 repeat in the fully methylated DNA sample)] × 100. A fully methylated DNA sample was prepared by treatment of genomic DNA with *SssI* methylase (New England Biolabs, Beverly, MA, USA). The differences of DNA methylation levels were evaluated by Mann–Whitney *U*‐test.

### Bisulfite sequencing using next‐generation sequencer

One microliter of bisulfite‐treated DNA was amplified using primers listed in Table S1, and the PCR product was purified using a Zymo‐Spin column I (Zymo Research). Using a purified PCR product, a DNA library was prepared using an Ion Fragment Library kit (Thermo Fisher Scientific, Waltham, MA, USA) and an Ion Xpress Barcode Adaptors 1‐96 kit (Thermo Fisher Scientific), and DNA libraries uniquely barcoded were pooled. The pooled library was mixed with Ion Spheres, and emulsion PCR was conducted using the Ion OneTouch 2 (Thermo Fisher Scientific) with an Ion PGM Template OT2 400 kit (Thermo Fisher Scientific). The emulsion PCR product was concentrated using the Ion OneTouch ES (Thermo Fisher Scientific) and loaded onto an Ion PI chip (Thermo Fisher Scientific). Sequencing was conducted using an Ion Proton sequencer (Thermo Fisher Scientific), as described previously.^20^ Methylation of 50% or less of CpG sites on a DNA molecule was defined as sparse DNA methylation, and that of 80% or more of CpG sites on a DNA molecule was defined as dense DNA methylation.

### Quantitative RT‐PCR

Total RNA was extracted from gastric tissues containing submucosal layers, isolated gastric epithelial cells, and gerbil gastric cancer cell lines using ISOGEN (Nippon Gene, Tokyo, Japan), and cDNA was synthesized using oligo‐dT primer (Thermo Fisher Scientific) and SuperScript III reverse transcriptase (Thermo Fisher Scientific). Synthesized cDNA was dissolved in 1× TE, and the copy number of individual genes was analyzed by quantitative PCR (RT‐qPCR), as described previously,^5^, ^19^ using primers listed in Table S1. The differences of gene expression levels were evaluated by Mann–Whitney *U*‐test.

## Results

### Inflammation activity according to infection period

**Figure 1:**
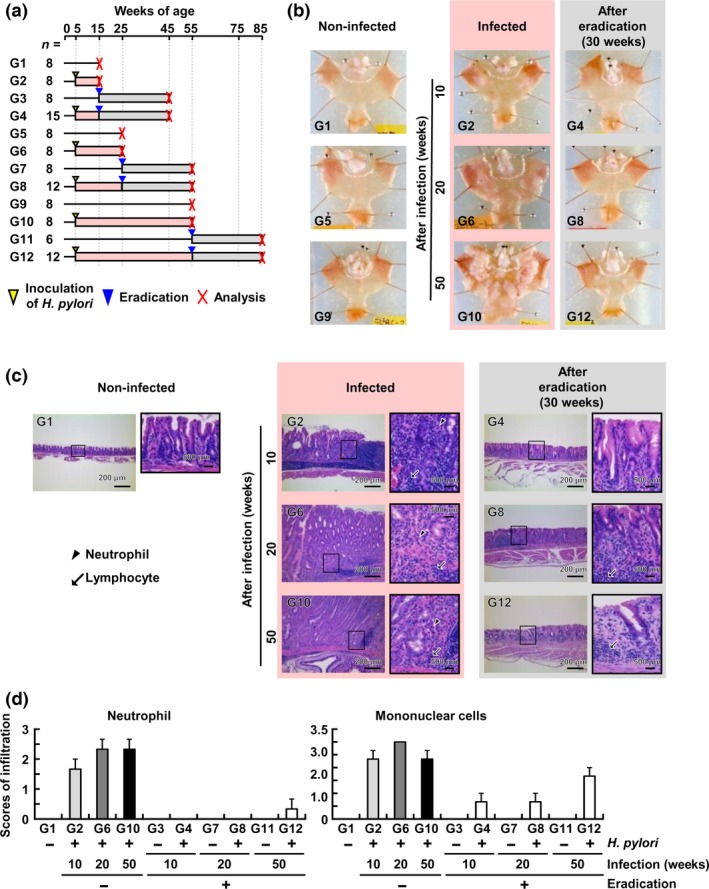
*Helicobacter pylori* infection period and the degree of chronic inflammation after eradication. (a) Experimental protocol of *H. pylori* infection and its eradication. Gerbils were infected with *H. pylori* for 10, 20, or 50 weeks, and analyzed before eradication and 30 weeks after eradication. (b) The *H. pylori* infection period and the degree of hyperplasia. The degree of hyperplasia was dependent upon the infection period, and was decreased after eradication. (c) The *H. pylori* infection period and the infiltration of inflammatory cells. Mononuclear cells were infiltrating independently of infection period, and their infiltration was reduced after eradication. (d) Quantification of infiltration of inflammatory cells. The degree of neutrophil or mononuclear cell infiltration was scored for 3 of the 6 (G11), 8 (G1, G2, G3, G6, G7, and G10), 12 (G8 and G12), and 15 (G4) gerbils on a four‐point scale (0 to 3), as described previously.^17^ In gerbils with 50 weeks of *H. pylori* infection, the infiltration of neutrophils and mononuclear cells remained, even 30 weeks after eradication. Mean score ± standard error (SE) is shown.

Macroscopic changes of the stomach according to different exposure period (infection period) were analyzed (Fig. 1a). The degree of hyperplasia was dependent upon the infection period (Fig. 1b), and was largest in gastric mucosae of gerbils infected for 50 weeks (Fig. 1b, G10). Microscopically, mononuclear cells and neutrophils were infiltrating, but the degree of the infiltration was similar among the gerbils with different infection periods (Fig. 1c,d).

After eradication, macroscopically, the degree of the hyperplasia decreased, but hyperplasia still remained in gastric mucosae of gerbils infected for 50 weeks (Fig. 1b, G4, G8, and G12). Microscopically, the infiltration of neutrophils was markedly reduced in gastric mucosae of gerbils infected for 10 and 20 weeks, but was still observed in gastric mucosae of gerbils infected for 50 weeks (Fig 1c,d). No infiltration of neutrophils was observed in those of age‐matched non‐infected gerbils. The infiltration of mononuclear cells was also reduced, but still remained in gastric mucosae of gerbils infected for 10, 20, and 50 weeks (Fig 1c,d).

**Figure 2:**
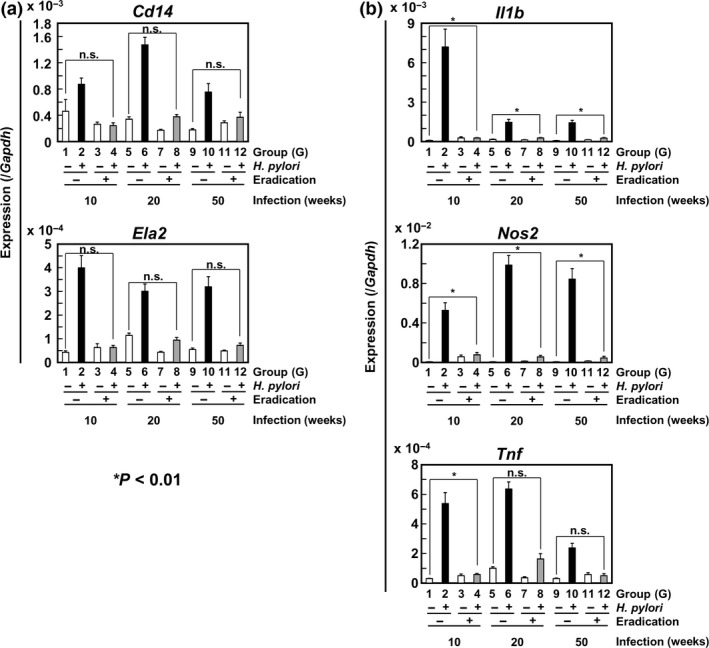
Expression levels of inflammatory cell markers (a) and inflammatory‐related genes (b) before and after eradication of *Helicobacter pylori*. Their expression levels were measured in entire gastric tissues by RT‐qPCR. Expression levels increased by *H. pylori* infection, but the influence of the infection period was not observed. After eradication, their expression levels were decreased, and the expression levels of the three inflammation‐related genes after eradication were not different among the gerbils with 10, 20, and 50 weeks of infection. Mean expression level ± standard error (SE) is shown.

We then analyzed mRNA expression levels of inflammatory cell markers, *Cd14* (macrophage) and *Ela2* (neutrophil), and inflammation‐related genes, *Il1b*,* Nos2*, and *Tnf*, which are known to be associated with aberrant DNA methylation induction.^10^, ^21^ Expression levels of inflammatory cell markers and inflammation‐related genes increased by *H. pylori* infection, but the levels were not increased according to the infection period (Fig. 2a,b). After eradication, expression levels of all the five genes decreased, but those of three inflammation‐related genes were significantly higher than those in gastric tissues of never‐infected gerbils (Fig. 2a,b). The expression levels of the three inflammation‐related genes after eradication were not different among the gerbils with 10, 20, and 50 weeks of infection (Fig. 2b).

### Increase of DNA methylation levels according to the infection period

**Figure 3:**
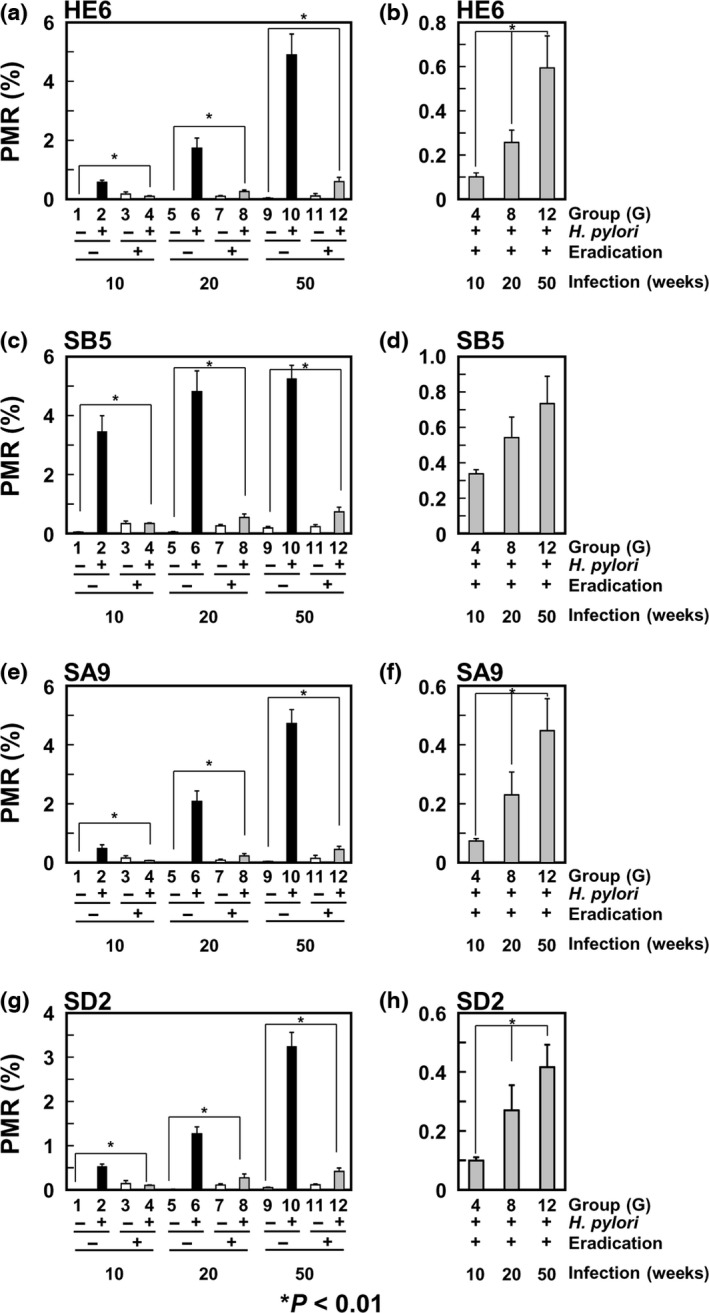
DNA methylation levels after eradication depending upon the *Helicobacter pylori* infection period. DNA methylation levels of the four CpG islands were analyzed in gastric mucosae by qMSP. Their DNA methylation levels increased according to the *H. pylori* infection period (a, c, e, and g). After eradication, DNA methylation levels decreased, but the degree of remaining DNA methylation tended to be higher in gastric mucosae of gerbils with longer infection periods (b, d, f, and h). Mean DNA methylation level ± standard error (SE) is shown.

To reveal the impact of the infection period on DNA methylation levels, methylation levels of four CpG islands were analyzed by qMSP because DNA methylation of specific genes is known to reflect the entire epigenome damage by *H*. *pylori* infection.^22^, ^23^ DNA methylation levels increased depending upon the infection period (Fig. 3a,c,e,g). DNA methylation levels of all the four CpG islands decreased after eradication, but were significantly higher than those in gastric mucosae of never‐infected gerbils (Fig. 3a,c,e,g). DNA methylation levels after eradication, which are considered to reflect epigenetic damage in stem cells (methylation burden),^24^ tended to be higher in gastric mucosae of gerbils with longer infection periods (Fig 3b,d,f,h).

To analyze the biological outcome of aberrant DNA methylation of these CpG islands, the association between methylation of SD2, a promoter CpG island of *Nptx2* gene, and *Nptx2* expression was analyzed. *Nptx2* expression was not detected in the gerbil gastric cancer cell lines, MGC1 and MGC2, with aberrant DNA methylation, but was detected in gastric mucosae of non‐infected gerbils without methylation (Fig. S1).

### Accumulation of dense DNA methylation according to infection period

**Figure 4:**
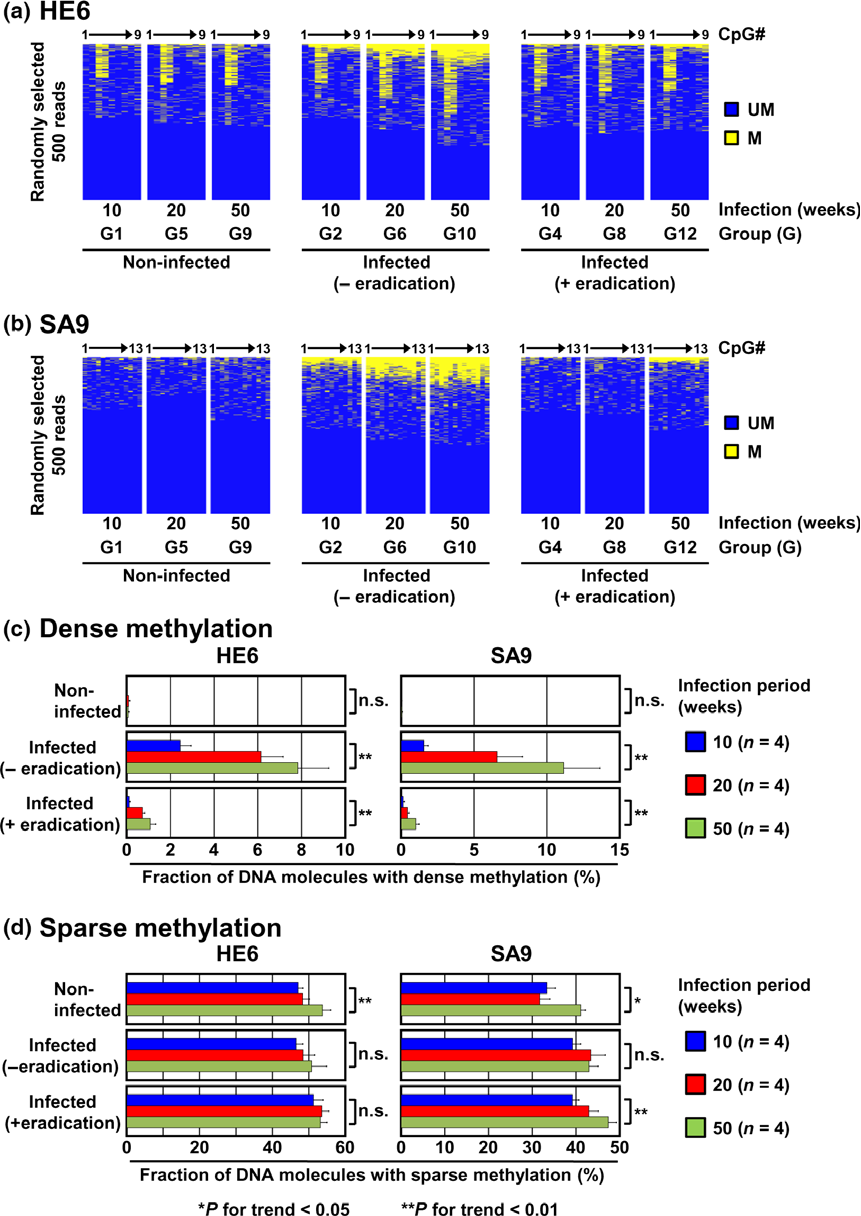
Accumulation of dense DNA methylation after eradication depending upon the *Helicobacter pylori* infection period. (a) and (b) DNA methylation status of HE6 and SA9. DNA methylation status was analyzed by bisulfite sequencing using a next‐generation sequencer. Number of methylated CpG sites after eradication increased depending upon the infection period. DNA methylation statuses of 500 DNA molecules randomly selected are shown. Yellow, methylated CpG sites; blue, unmethylated CpG sites. (c) and (d) Fraction of DNA molecules with dense (c) and sparse (d) DNA methylation. Fraction of dense methylation increased depending upon the infection period. Mean fraction ± standard error (SE) is shown.

Sparse methylation of a CpG island cannot cause transcriptional silencing of its downstream gene, but is important to induce dense DNA methylation of the CpG island, which can cause transcriptional silencing.^25^, ^26^, ^27^ Therefore, dynamics of sparse and dense DNA methylation according to different infection periods were analyzed for HE6 and SA9 by bisulfite sequencing combined with next‐generation sequencing. The fraction of dense methylation (methylation of 80% or more of CpG sites on a DNA molecule) increased depending upon the *H*. *pylori* infection period before eradication (Fig. 4a–c). After *H*. *pylori* eradication, the fraction of dense methylation decreased, but was significantly higher than that in gastric mucosae of never‐infected gerbils (Fig. 4c). The fraction of dense methylation after eradication was larger in gastric mucosae of gerbils with longer infection periods (Fig. 4c). Also by aging, the fraction of dense methylation increased, but the age‐dependence was unclear (Figs 4c and S2).

The fraction of sparse methylation (methylation of 50% or less of CpG sites on a DNA molecule) was around 30 to 50%, even in gastric mucosae of non‐infected gerbils. The fraction of sparse methylation slightly increased by *H*. *pylori* infection only for SA9, but was not decreased even after eradication (Fig. 4a,b,d). In contrast, the fraction of sparse methylation increased by aging in an age‐dependent manner (Fig. 4d). These results indicated that accumulation of dense DNA methylation but not sparse DNA methylation after *H*. *pylori* eradication increased depending upon the infection period.

### Long‐term persistence of aberrant DNA methylation after *H. pylori* eradication

**Figure 5:**
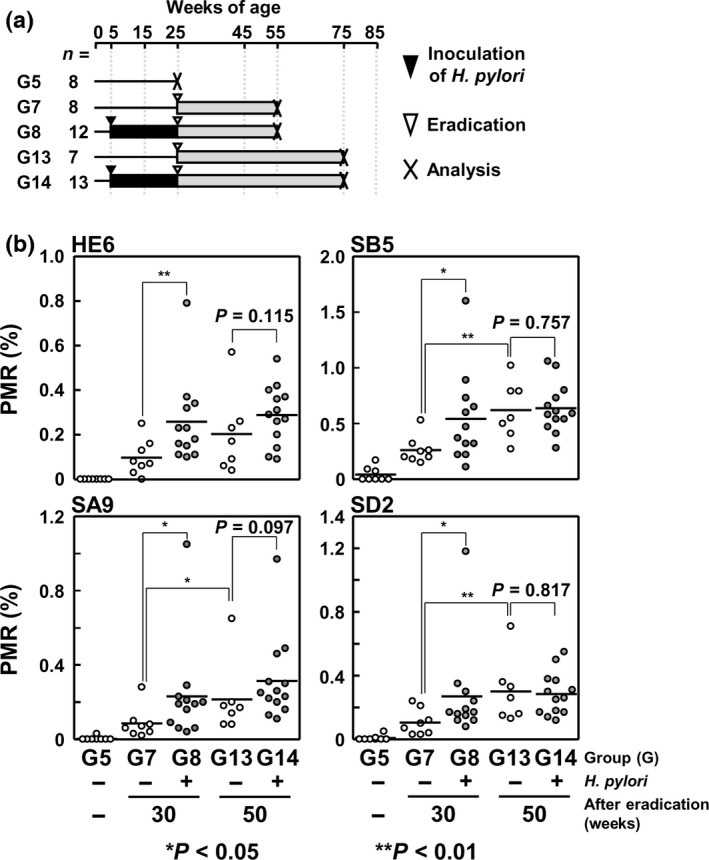
Long‐term persistence of aberrant DNA methylation after eradication. (a) Experimental protocol of *Helicobacter pylori* infection and its eradication. Gerbils were infected with *H. pylori* for 20 weeks, and DNA methylation levels were analyzed 30 and 50 weeks after eradication. (b) DNA methylation levels after *H. pylori* eradication. DNA methylation levels in gastric mucosae were analyzed by qMSP. At 30 weeks after eradication, DNA methylation levels of the four CpG islands were higher in gastric mucosae of infected gerbils than in those of non‐infected gerbils. Even at 50 weeks after eradication, one of the four CpG islands (SA9) tended to be higher in gastric mucosae of infected gerbils than in those of non‐infected gerbils, but the difference was not statistically significant.

To reveal whether or not DNA methylation remains for a long period after *H*. *pylori* eradication, DNA methylation levels in gastric mucosae were analyzed at 30 and 50 weeks after eradication (Fig. 5a). At 30 weeks after eradication, DNA methylation levels of all the four CpG islands were significantly higher in gastric mucosae of *H*. *pylori*‐infected gerbils (Fig. 5b, G8) than in those of non‐infected gerbils (Fig. 5b, G7). In contrast, at 50 weeks after eradication, DNA methylation levels of only one CpG island (SA9) tended to be higher in gastric mucosae of *H*. *pylori*‐infected gerbils (Fig. 5b, G14) than in those of non‐infected gerbils (Fig. 5b, G13), but the difference was not statistically significant (*P *=* *0.097). This result indicated that aberrant DNA methylation could persist for a long period after *H. pylori* eradication.

## Discussion

Persistent aberrant DNA methylation after *H*. *pylori* eradication, namely methylation burden, increased depending upon the infection period. This showed for the first time that the levels of methylation burden in normal tissues are influenced by the degree of exposure to a carcinogenic factor (inducers of aberrant DNA methylation). Based on the finding, it was suggested that elimination of the exposure to carcinogenic factors, such as the eradication of *H*. *pylori* in gastric tissues, at its early stage is beneficial for cancer prevention. This finding was obtained by the utilization of an excellent animal model, namely a Mongolian gerbil model of *H*. *pylori*‐induced gastritis, in which aberrant DNA methylation can be efficiently induced.^10^, ^11^

Dense DNA methylation, but not sparse DNA methylation, after *H*. *pylori* eradication increased depending upon infection period. This suggested that the molecular mechanisms are different for induction of dense and sparse DNA methylation. Specifically, sparse methylation is likely to be induced by the dysregulation of methylation machineries, such as upregulation of DNA methyltransferases or downregulation of ten‐eleven translocation (Tet) hydroxylases, which is involved in active DNA demethylation.^28^ Dense methylation might be, then, induced by conversion of sparse methylation during cell replication. To show this possible mechanism, it is required to analyze effects of the dysregulation of methylation machineries and cell replication on the changes of DNA methylation status.

At 30 weeks after *H. pylori* eradication, DNA methylation levels of all the four CpG islands in gastric mucosae of gerbils infected with *H*. *pylori* were significantly higher than in those of non‐infected gerbils. In contrast and unexpectedly, at 50 weeks after eradication, DNA methylation levels of only one of the four CpG islands tended to be higher, but the difference was not statistically significant. This was considered to be due to the increase of DNA methylation levels in non‐infected gerbils by aging. Based on this finding, it was suggested that selection of marker genes is important to conduct accurate assessment of the methylation burden. Also, in humans, the importance of marker selection was shown for the risk assessment of human gastric carcinogenesis.^23^

In conclusion, the amount of methylation burden in normal tissues is determined by the degree of exposure, such as the exposure period, to a carcinogenic factor, and the importance of elimination of such a factor at any time was suggested.

## Disclosure Statement

The authors have declared that no competing interests exist.

## Supporting information


**Fig S1.** The association between SD2 methylation and *Nptx2* expression. (a) DNA methylation status of SD2 in gastric mucosae of non‐infected gerbils and gerbil gastric cancer cell lines, MGC1 and MGC2. SD2 was not methylated in normal gastric mucosae, but aberrantly methylated in the cancer cell lines. M, primers specific to methylated DNA; U, primers specific to unmethylated DNA. (b) Expression levels of *Nptx2* in gastric mucosae of non‐infected gerbils and gerbil gastric cancer cell lines. *Nptx2* expression was not detected in gerbil gastric cancer cell lines with SD2 methylation while its expression was detected in gastric mucosae of non‐infected gerbils without methylation.

**Fig. S2.** Magnification of Fig. 4c. Fraction of DNA molecules with dense DNA methylation in gastric mucosae of non‐infected gerbils is shown. Mean fraction ± standard error (SE) is shown.

**Table S1**. Primers used for DNA methylation analysis and expression analysis.
