# Supplementary figures and images for: Degree of methylation burden is determined by the exposure period to carcinogenic factors
Source: Cancer Sci. 2017 Apr 3;108(3):316–21. doi: 10.1111/cas.13136 (PMC5378290; doi:10.1111/cas.13136)

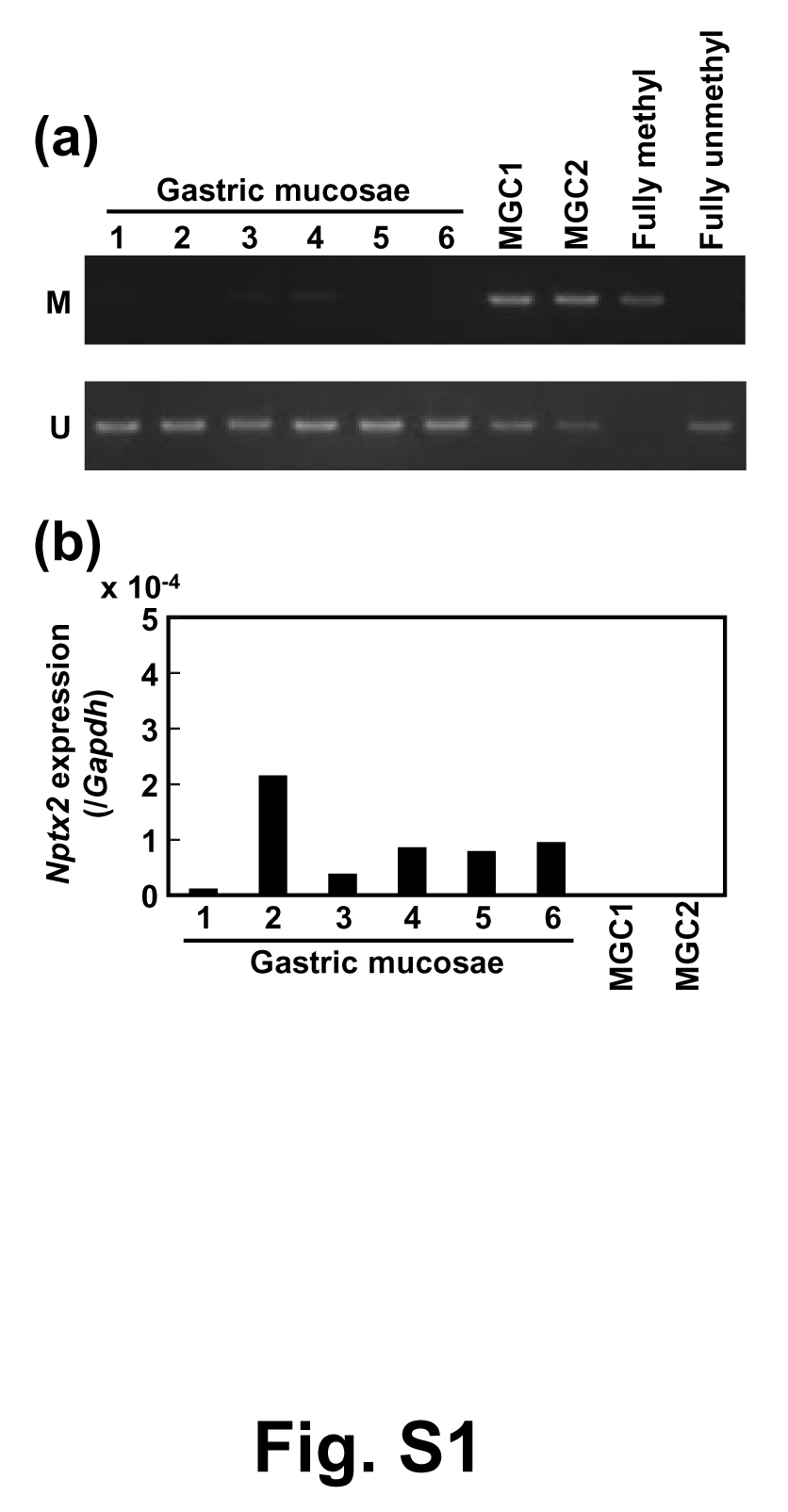

Supplement: Supplementary file 1 — Fig S1. The association between SD2 methylation and Nptx2 expression. (a) DNA methylation status of SD2 in gastric mucosae of non‐infected gerbils and gerbil gastric cancer cell lines, MGC1 and MGC2. SD2 was not methylated in normal gastric mucosae, but aberrantly methylated in the cancer cell lines. M, primers specific to methylated DNA; U, primers specific to unmethylated DNA. (b) Expression levels of Nptx2 in gastric mucosae of non‐infected gerbils and gerbil gastric cancer cell lines. Nptx2 expression was not detected in gerbil gastric cancer cell lines with SD2 methylation while its expression was detected in gastric mucosae of non‐infected gerbils without methylation. [file CAS-108-316-s001.tif]

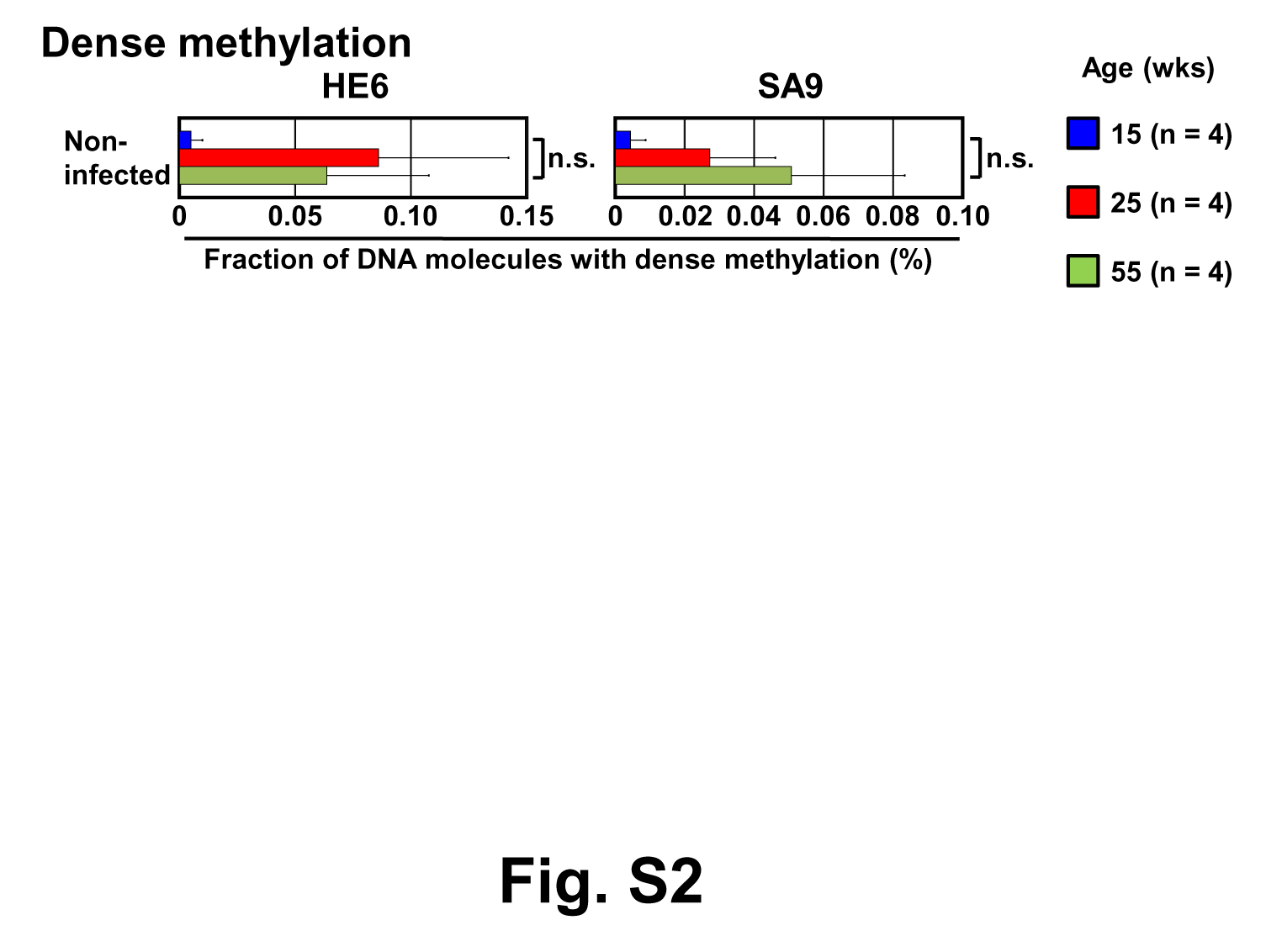

Supplement: Supplementary file 2 — Fig. S2. Magnification of Fig. 4c. Fraction of DNA molecules with dense DNA methylation in gastric mucosae of non‐infected gerbils is shown. Mean fraction ± standard error (SE) is shown. [file CAS-108-316-s002.tif]
