# Supplementary material for: Degree of methylation burden is determined by the exposure period to carcinogenic factors
Source: Cancer Sci. 2017 Apr 3;108(3):316–21. doi: 10.1111/cas.13136 (PMC5378290; doi:10.1111/cas.13136)
Supplement: Supplementary file 3 — Table S1. Primers used for DNA methylation analysis and expression analysis. [file CAS-108-316-s003.pdf]

Table S1. Primers used for DNA methylation analysis and expression analysis.

Gene	Type	Forward primer sequence	Reverse primer sequence	Annealing temperature (ºC)	Reference
HE6	M	CGGTTGGGACGTCGCGGGTCGTC	CTAAAATACGCACCGATAACCGCG	72	1)
SB5	M	GGGGATCGATTTTTAACGTTCGC	CAAACTCAAACCGCCACGTCCG	72	1)
SA9	M	GATTCGTAAGAAATACGAGCGGATC	AACGTTATCGATAAAACTCGCCTCG	72	1)
SD2	M	GCGCGGGGTTTTCGAGGTTC	GCCATCACCTCCCATCGTAAACG	72	1)
SD2	U	TGTGAGTGTGGGGTTTTTGAGGTTT	ACACCATCACCTCCCATCATAAACA	62	1)
B2 repeat	N	AGATGGTTGTGAGTTATTATGTG	CTATTCTTCCAAAAATCCTAAAT	51	1)
HE6	BS	GTATTTTAGGGTTGAGGATAAATAGA	AAACACTCAAACATCCCTTC	60	This study
SA9	BS	AGGATTGGTGTTTTAGGATTT	CTAACCCAACTAAAAACCCTTC	54	This study
Cd14	RT	TGTCCCAACAAGTTTCCGGCACT	TCCAGTTCCTGCGGCTGTACCTT	69	1)
Cd3g	RT	CGAGGCGTGTTTCAGTGTCAAGG	GCGAAGAAGAAGCCGAATATGGT	54	1)
Ela2	RT	GCTACCATCAACGCCAACGTG	GCTGGGTGAGGGTCGGTTTGTGC	69	1)
Il1b	RT	AACAGTGGGCAGGATATAGTTGA	CCCATCTTGGCAGAGGACTAAGG	66	1)
Nos2	RT	CGAGCCCAAGGTCTACGTCCAA	CGCACGTCTCCGCAGACATAGAG	60	1)
Tnf	RT	GCATGGAGCTCAGGGACAACCAG	GGCTGACGGTGTGCGTGAGGA	69	1)
Nptx2	RT	CCGCGCCATCACCTCCCATCGT	AAAGCGGCCACAGAAAGCGTTG	62	1)

M, primers specific to methylated DNA; U, primers specific to unmethylated DNA; N, primers for normalization of qMSP; BS, primers for deep bisulfite sequencing; RT, primers for quantitative RT-PCR; and 1) Niwa et al., Cancer Res, 70:1430-1440, 2010.
